# Diagnosis, misdiagnosis, lucky guess, hearsay, and more: an ontological analysis

**DOI:** 10.1186/s13326-016-0098-5

**Published:** 2016-09-15

**Authors:** William R. Hogan, Werner Ceusters

**Affiliations:** 1 University of Florida, 2004 Mowry Rd, P.O. Box 100219, Gainesville, FL 32610-0219 USA; 2 Department of Biomedical Informatics, University at Buffalo, 77 Goodell street, 5th floor, Buffalo, NY 14203 USA

**Keywords:** Biomedical ontology, Referent tracking, Disease, Diagnosis, Information content entity, Representation, Ontological realism

## Abstract

**Background:**

Disease and diagnosis have been the subject of much ontological inquiry. However, the insights gained therein have not yet been well enough applied to the study, management, and improvement of data quality in electronic health records (EHR) and administrative systems. Data in these systems suffer from workarounds clinicians are forced to apply due to limitations in the current state-of-the art in system design which ignore the various types of entities that diagnoses as information content entities can be and are about. This leads to difficulties in distinguishing amongst diagnostic assertions misdiagnosis from correct diagnosis, and the former from coincidentally correct statements about disease.

**Methods:**

We applied recent advances in the ontological understanding of the aboutness relation to the problem of diagnosis and disease as defined by the Ontology for General Medical Science. We created six scenarios that we analyzed using the method of Referent Tracking to identify all the entities and their relationships which must be present for each scenario to hold true. We discovered deficiencies in existing ontological definitions and proposed revisions of them to account for the improved understanding that resulted from our analysis.

**Results:**

Our key result is that a diagnosis is an information content entity (ICE) whose concretization(s) are typically about a configuration in which there exists a disease that inheres in an organism and instantiates a certain type (e.g., hypertension). Misdiagnoses are ICEs whose concretizations succeed in aboutness on the level of reference for individual entities and types (the organism and the disease), but fail in aboutness on the level of compound expression (i.e., there is no configuration that corresponds in total with what is asserted). Provenance of diagnoses as concretizations is critical to distinguishing them from lucky guesses, hearsay, and justified layperson belief.

**Conclusions:**

Recent improvements in our understanding of aboutness significantly improved our understanding of the ontology of diagnosis and related information content entities, which in turn opens new perspectives for the implementation of data capture methods in EHR and other systems to allow diagnostic assertions to be captured with less ambiguity.

**Electronic supplementary material:**

The online version of this article (doi:10.1186/s13326-016-0098-5) contains supplementary material, which is available to authorized users.

## Background

As administrative, clinical, and patient-reported data are increasingly shared and reused, especially for patient care [1–4] and research [1, 5–7], several issues with these data—including diagnosis data—are of increasing concern. The issue that appears to be of greatest concern is data error and the implications thereof for making decisions and conclusions based on them [8–13]. Although Shapiro et al., in a report for the Office of the National Coordinator for Health Information Technology, do not cite error as a concern for including patient-generated health data into the electronic health record (EHR) [14], there are known errors with patient self reporting especially in research [15–22]. A second issue of concern is data provenance [10, 23], i.e. information about who created the data, in what setting, how, when, for what purpose, and so on. For example, Johnson et al. noted that the provenance of symptom data was essential to using those data correctly to determine whether a colonoscopy was a screening vs. diagnostic procedure [23].

Data error and data provenance are closely related. For example, Hersh et al. note that data recorded in billing workflows for financial purposes are less accurate than clinical data [10]. Thus, timing, method, and purpose of recording data at a minimum—all aspects of provenance—are intertwined with accuracy. Furthermore, a key result of the Johnson et al. study is that “Researchers who do not consider data provenance risk compiling data that are systematically incomplete or incorrect*”* [23].

An ontological account of data error and data provenance can identify crucial distinctions. For example, there are significant differences among (1) a measured weight that is off because the scale was not properly tared, (2) a ‘rough’ weight of 70 kg entered in an emergency when the patient cannot be weighed, and (3) a weight measurement entered on the wrong patient. Detecting and accounting for these differences and their causes—especially the aspects of provenance that influence them—is necessary to inform strategies to study, cope with, and improve data error when using pre-existing EHR data for research.

Additionally, a recent review article on the methods for assessing quality of EHR data for clinical research found that: *Most of the studies included in this review presented assessment methodologies that were developed with a minimal empirical or theoretical basis* [24]. It concluded with a call for moving away from ad hoc approaches to data quality assessment, to formal, validated approaches. Although error is only one aspect of data quality (fitness for purpose and completeness are two others), a formal ontological understanding of data error could play a role in more formalized methods for data quality assessment.

In this work, we apply Smith and Ceusters’ recent ontological account of incorrect information (i.e., error) [25] to diagnosis data in administrative systems, EHRs, and patient-reported information. Their account holds that a statement such as a diagnostic assertion can succeed or fail in aboutness on at least two levels: (1) the level of denoting single entities and/or types (i.e., the level of *reference*) and (2) the level of veridical representation of a configuration of multiple entities and/or types (i.e., the level of *compound expression*).

To succeed on the second level (compound expression), the information content entity (ICE) must be correct about *all* particulars, their relationships, and their instantiations of types that it mentions. Failure on a single particular, relation, or instantiation causes the ICE to fail at the second level while still potentially succeeding at the first level. For example, if Mrs. Jones has type 1 diabetes mellitus, then the sentence ‘*Mrs. Jones suffers from type 2 diabetes mellitus’* fails in aboutness on the level of compound expression because it misstates one thing: her disease does not instantiate type 2 diabetes mellitus. However, despite this failure the sentence is nevertheless still about Mrs. Jones, about her disease, and about type 2 diabetes mellitus on the level of reference, because indeed it mentions those three entities. It is therefore, per Smith and Ceusters, an ICE that is about *something* even though it is a misdiagnosis.

Prior ontological work on the aboutness of clinical statements like diagnoses has been constrained by the view that an ICE is about nothing (or is perhaps not even an ICE at all) if it fails on the level of compound expression. Martínez Costa and Schulz, for example, use the universal quantifier when relating an information entity to a clinical situation *…to avoid asserting the existence of an entity the existence of which cannot be guaranteed* [26]. For an ICE such as ‘suspected heart failure’ they want to avoid the implication that there is some instance of heart failure that it is about. Because they cannot guarantee the existence of some heart failure, they use universal quantification to say ‘if it is about anything, then it is about an instance of heart failure’. Researchers working in areas other than diagnosis have encountered similar issues. For example, Hastings et al. note that chemical graphs and diagrams are not always about types of molecules that exist [27]. They, too, used the workaround of replacing existential quantification with universal quantification to avoid asserting that every chemical graph/diagram is about some type of molecule that exists (level of compound expression), while still allowing such graphs and diagrams to be subtypes of information content entity.

In our own, previous ontological analysis of diagnosis, using the methodology of referent tracking, we identified what entities must exist or must have existed for a particular diagnostic statement to hold true [28, 29]. A key result of this work is that a diagnosis is minimally about *both* the patient and the type of disease that is asserted to exist. In addition, building on previous work on the Ontology for General Medical Science (OGMS), the foundations of which were laid down in Scheuermann et al. [30], we noted that for a diagnosis to exist (at least in medicine and under the assumption that the diagnosis was made *lege artis*), there must also have existed a diagnostic process, a person who carried out that process, and a clinical picture which was used as input into that process.

The hypothesis for the work described here was that applying Smith and Ceusters’ results to disease and diagnosis, in combination with prior work on the ontology of disease and diagnosis (including provenance of the latter), could address limitations encountered in previous ontological work on disease and diagnosis and improve our representations of them in support of studying, coping with, and reducing ambiguity in the generation of diagnostic statements and error in the interpretation thereof.

## Methods

To test this hypothesis, we analyzed a set of scenarios that we created and that involve correct and incorrect diagnoses, lucky guesses, and justified layperson belief in the existence of a disease of a certain type. The goal was to explore whether, and if so how, a realism-based account of information can deal successfully not only with diagnostic statements asserting the ideal case of a correct diagnosis, but also with deviations from the ideal.

### Materials

In our analysis we used as input (1) Smith and Ceusters’ work on aboutness and their definitions of representation, mental quality, cognitive representation, and information quality entity (Table 1), (2) definitions of disease, disorder, and diagnosis from the Ontology for General Medical Science (Table 2), and (3) our prior work on analysis of diagnostic statements [27, 28].

**Table 1:** Definitions based on Smith and Ceusters [25]

Term	Definition
INFORMATION CONTENT ENTITY	An ENTITY which is (1) GENERICALLY DEPENDENT on (2) some MATERIAL ENTITY and which is (3) concretized by a QUALITY (a) inhering in the MATERIAL ENTITY and (b) that is_about some PORTION OF REALITY
INFORMATION QUALITY ENTITY	A REPRESENTATION that is the concretization of some INFORMATION CONTENT ENTITY
REPRESENTATION	A QUALITY which is_about or is intended to be about a PORTION OF REALITY
MENTAL QUALITY	A QUALITY which specifically depends on an ANATOMICAL STRUCTURE in the cognitive system of an organism
COGNITIVE REPRESENTATION	A REPRESENTATION which is a MENTAL QUALITY
Relation	Explanation
*x is_about y*	*x refers to or is cognitively directed towards y*. Domain: representations; Range: portions of reality
*x concretizes y*	*x* is a QUALITY and *y* is a GENERICALLY DEPENDENT CONTINUANT (GDC) and for some MATERIAL ENTITY *z*, *x specifically_depends_on* z at *t* and *y generically_depends_on z* at *t*, and if *y* migrates from bearer *z* to another bearer *w* then a copy of *x* will be created in *w*.
*x is_conformant_to y*	=def. *x* is an INFORMATION QUALITY ENTITY and *y* is a COGNITIVE REPRESENTATION and there is some GDC *g* such that *x concretizes g* and *y concretizes g.*

**Table 2:** Key definitions from OGMS used in the analysis

Term	Definition
DISEASE	A DISPOSITION (i) to undergo PATHOLOGICAL PROCESSes that (ii) exists in an ORGANISM because of one or more DISORDERs in that ORGANISM.
DISORDER	A causally relatively isolated combination of physical components that is (a) clinically abnormal and (b) maximal, in the sense that it is not a part of some larger such combination.
DIAGNOSIS	A conclusion of an interpretive PROCESS that has as input a CLINICAL PICTURE of a given patient and as output an assertion (diagnostic statement) to the effect that the patient has a DISEASE of such and such a type.
DIAGNOSTIC PROCESS	An interpretive PROCESS that has as input a CLINICAL PICTURE of a given patient and as output an assertion to the effect that the patient has a DISEASE of a certain type.
PATHOLOGICAL PROCESS	A bodily PROCESS that is a manifestation of a DISORDER.
PHENOTYPE	A bodily feature or combination of bodily features of an organism determined by the interaction of the genetic make-up of the organism and its environment.
CLINICAL PHENOTYPE	A clinically abnormal PHENOTYPE.
CLINICAL PICTURE	A representation of a CLINICAL PHENOTYPE that is inferred from the combination of laboratory, image and clinical findings about a given patient.
CLINICAL FINDING	A REPRESENTATION that is either the output of a clinical history taking or a physical examination or an image finding, or some combination thereof.
MANIFESTATION OF DISEASE	A QUALITY of a patient that is (a) a deviation from clinical normality that exists in virtue of the realization of a disease and (b) is observable.
CLINICAL HISTORY TAKING	An interview in which a clinician elicits a clinical history from a patient or from a third party who is authorized to make health care decisions on behalf of the patient.
CLINICAL HISTORY	A series of statements representing health-relevant features of a patient.

Smith and Ceusters stressed that the relation of aboutness includes any portion of reality, rather than being limited to just a single particular or a single universal. A portion of reality (POR) can be a particular, a universal, a relation, or a configuration. A configuration is a combination of particulars and/or universals and certain relation(s) that hold among them.

A representation, then, that is intended to be about a POR but fails in its aboutness because it misrepresents that POR in some way, is misinformation. The sentence *Bob Dylan was in the Beatles* fails to represent not because Bob Dylan or the Beatles did not exist, but because such a configuration involving Bob Dylan and the Beatles in the way as expressed, never existed. The sentence fails in aboutness on the level of compound expression, but nevertheless is about Bob Dylan and the Beatles individually (on the level of reference) and thus is still an information content entity.

Smith and Ceusters [25] deal more fully with the issue of what it means that a representation is “intended to be about” some entity. Here, we highlight that it follows the doctrine of the “primacy of the intentional” [31], where our written and verbal expressions are to be understood on the basis of the cognitive acts that generated them. That is, a sentence is about that to which its author was directing his or her thoughts when she wrote it.

In addition to Smith and Ceusters’ work, we also founded our ontological analysis on the Ontology for General Medical Science or OGMS [30]. This work distinguishes disease, disorder, and diagnosis, and we used definitions from OGMS as starting points for our analysis (Table 2). Note that in OGMS, a diagnosis refers to the existence of a disease of a given type. In clinical medicine, however, diagnoses also refer to (1) disease courses (e.g., acute hepatitis vs. chronic hepatitis), (2) disorders (e.g., fractures and tumors), and (3) the absence of any disease (i.e., a conclusion that a person is healthy also is a diagnosis). It was not our goal to address this issue in this work, as it was not our goal to refine the OGMS definition of diagnosis.

### The scenarios

**Fig. 1:**
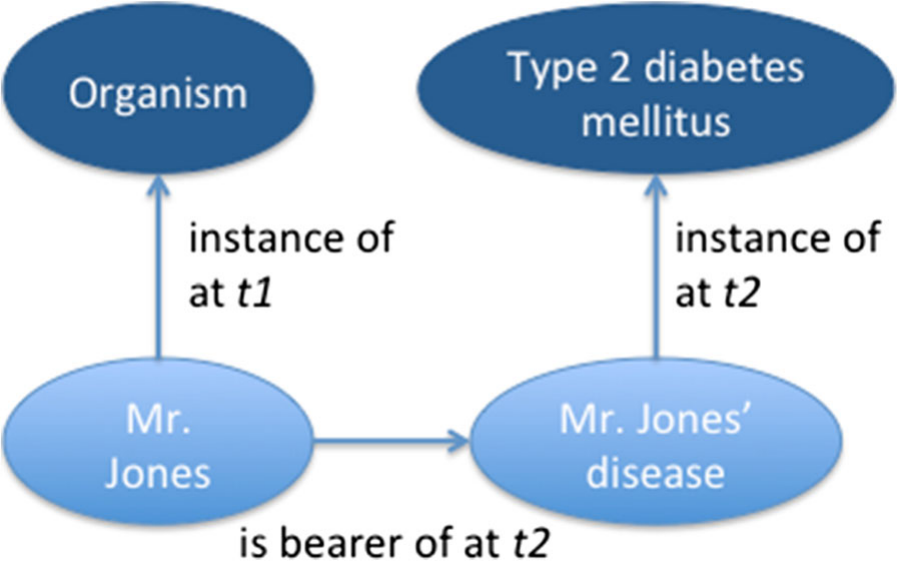
The configuration of Mr. Jones, his disease, and type 2 diabetes mellitus

All the scenarios have in common a particular patient, Mr. Adam Jones, who suffers from type 2 diabetes mellitus. Thus in every scenario, there exists Mr. Jones, his disease, the type *Type 2 diabetes mellitus*, the configuration of these three entities (which includes the “bearer of” and “instance of” relationships), and the placement in space and time of this configuration (Fig. 1).

#### Scenario 1: correct diagnosis by physician (ideal case)

Dr. Anne Smith sees Mr. Jones in the office. She takes a history and physical, performs certain laboratory testing, and based on her analysis of the findings, correctly concludes that Mr. Jones has type 2 diabetes mellitus. She subsequently writes her diagnosis in the patient’s medical record.

#### Scenario 2: subsequent correct diagnosis by physician using first diagnosis

A second doctor, Dr. John Brown, sees Mr. Jones in the office at some later date. Mr. Jones has released his records from Dr. Smith to Dr. Brown, who subsequently sees Dr. Smith’s diagnosis prior to seeing Mr. Jones. He uses that diagnosis plus his own findings to infer a new clinical picture of Mr. Jones, which he subsequently uses to make another correct diagnosis of Mr. Jones’ disease. He writes his diagnosis in Mr. Jones’ medical record.

#### Scenario 3: incorrect diagnosis by physician

Mr. Jones is traveling on vacation, when he falls ill. He sees Dr. Jane Miller who does not have any of his past records available, and thus she is not aware of the previous diagnoses of Drs. Smith or Brown. She infers a new clinical picture of Mr. Jones, and based on it incorrectly concludes that Mr. Jones has *type 1 diabetes mellitus* (as opposed to type 2). She records a diagnosis of type 1 diabetes mellitus in her medical record for for Mr. Jones.

#### Scenario #4: coincidentally correct conclusion by layperson (lucky guess)

A friend of Mr. Jones is a “seer”. Mr. Jones asks his friend what is in his future. Having no prior knowledge of Mr. Jones medical conditions, the “seer” concludes based on Mr. Jones’ horoscope and the position of the moon that he has type 2 diabetes mellitus. He subsequently predicts that Mr. Jones will be hospitalized for his diabetes and miss his daughter’s wedding.

#### Scenario #5: layperson’s justifiable conclusion

Mr. Jones’ daughter, upon learning of her father’s type 2 diabetes mellitus, adds this information into her letter to her brother, writing “Dad has type 2 diabetes mellitus”.

#### Scenario #6: correct diagnosis by computer-based expert system

A medical student is seeing Mr. Jones in the clinic. He performs a history and physical, and types his findings into a diagnostic expert system. The diagnostic expert system infers based on these findings that Mr. Jones has type 2 diabetes mellitus. The medical student writes this diagnosis in Mr. Jones’ medical record.

### The analysis

Our analysis follows the method of Referent Tracking, which we have found to be a stringent test of ontologies and their definitions [28]. This approach proceeds in three main steps. First, we systematically identify all the relevant particulars that must exist for the scenario to be true, regardless of whether the scenario explicitly mentions them or only implies their existence. We assign each particular an instance unique identifier (IUI), of the form ‘IUI-n’, where ‘n’ is any integer. Second, we identify for each particular the type it instantiates and the temporal interval during which it exists (and assign an identifier of the form *tn* to that interval). Lastly, we identify the relationships that hold between the particulars as well as all relevant relations particulars have to universals other than instantiation, including situations where a particular lacks a given relation to any instance of a certain type (for example, a statement that a patient has had no cough in the last two weeks means that the patient does not stand in the agent_of relation to any instance of the type *Coughing event*, indexed temporally to the two-week interval) [32].

This approach identifies problems in ontologies and their definitions in two major ways. First, it identifies problems that occur when the scenario explicitly rules out the existence of a particular whose existence is implied by an ontological definition (and vice versa). Second, it helps identify exceptions to existing definitions and situations that should not fall under a definition but are erroneously captured by it. Definitions in ontologies can subsequently be adjusted to avoid the errors so identified.

Although our approach is to identify particulars implied by sentences in natural language, the ontological analysis of language and the mechanism(s) by which it makes implicit reference to certain entities is not the focus of this work. Therefore, we convert a sentence like “Mr. Jones has type 2 diabetes mellitus” to Referent Tracking Tuples (e.g., as in Tables 3, 4, 5, 6 and 7) and it is these tuples in which inhere representations that are the objects of our analysis.

**Table 3:** Referent tracking tuples true in every scenario

IUI	Entity	Existence period	Type	Notes
IUI-1	Mr. Adam Jones	*t1* – the period during which IUI-1 exists	Material Entity	
IUI-2	IUI-1’s disease	*t2*	Disposition	
Relationships among particulars				
IUI-2	inheres in	IUI-1	at *t2*	
IUI-2	instance of	UUI-1	at *t2*	UUI-1 is a universal unique identifier that denotes *type 2 diabetes mellitus.* We assume that if something is at any time of its existence an instance of type 2 DM, it is instance of type 2 DM at all times it exists.

**Table 4:** The entities in Scenario 1

IUI	Entity	Existence period	Type	Notes
IUI-3	Dr. Anne Smith	t3	Human being	
IUI-4	Cognitive system of IUI-3	t4		
IUI-5	An anatomical entity that is part of IUI-4	t5	Anatomical entity	Which anatomical entity and its lifetime cannot be easily specified given current state of neuroscience.
IUI-6	Quality that inheres in IUI-5 and is about IUI-7	t6	Cognitive representation	
IUI-7	The POR that is truth-maker for IUI-8	t7	Configuration	Mr. Jones, his disease, their relationship, and disease’s instantiation
IUI-8	Dr. Smith’s diagnosis	t8	Diagnosis	ICE concretized by IUI-6 and IUI-10
IUI-9	That which is written down on paper and forms the sentence.	t9	Material entity	*I conclude therefore that Mr. Jones has type 2 diabetes mellitus.*
IUI-10	IQE that inheres in IUI-9.	t10	Information quality entity	The sentence began to exist as soon as ink was laid down on paper, but the IQE did not begin to exist until the sentence was finished.
IUI-11	Dr. Smith’s interpretive process	occupies t11	Diagnostic process	Dr. Smith’s diagnostic process that led to her diagnosis IUI-8
IUI-12	The clinical picture input into IUI-11	t12	Clinical picture	Dr. Smith’s clinical picture as ascertained prior to t6
IUI-13	Dr. Smith writing her diagnosis in the note	occupies t13	Process

**Table 5:** Additional temporal entities in Scenario 1

Temporal identifier	Description	Notes
t14	The interval during which the anatomical entity (IUI-5) is part of the cognitive system (IUI-4)	This interval is not easily specified given the current state of neuroscience. It could be different than t3 and t4.
t15	The interval during which the clinical picture (IUI-12) is used in the interpretive process (IUI-11)	Could be shorter than t11
t16	The point in time at which the cognitive representation (IUI-6) and diagnosis (IUI-8) begin to exist	t16 ends t11. Because the ICE does not exist until the cognitive representation—its first concretization—exists, this is also the point in time at which the diagnosis begins to exist.
t17	The interval during which the cognitive representation (IUI-6) participates in the writing process (IUI-13)	
t18	The interval during which the diagnosis (IUI-8) participates in the writing process (IUI-13)	It is possible that the original cognitive representation (IUI-6) gets copied elsewhere in the brain for reasoning and thus that the ICE continues to participate after the initial cognitive representation
t19	The interval during which that which is written on paper (IUI-10) begins to exist until it exists in full	The writing process begins earlier than the time at which the sentence begins to exist: the author starts the process with getting a pen and paper, any preparation necessary (“clicking” the pen), etc.

**Table 6:** Relationships among particulars in Scenario 1

IUI	Relation	IUI	When relation holds in reality	Notes
IUI-4	part of	IUI-3	at t4	
IUI-5	part of	IUI-4	at t14	All anatomical components in which the cognitive representation inheres are part of the cognitive system. We do not assume the cognitive system is limited to the brain, as the state of neuroscience does not permit such an assumption.
IUI-6	inheres in	IUI-5	at t6	
IUI-6	is about	IUI-7	at t6	The cognitive representation stands in aboutness to IUI-7 as long as it exists
IUI-6	is about	IUI-1	at t6	It is also about Mr. Jones
IUI-6	is about	IUI-2	at t6	And about Mr. Jones’ disease
IUI-6	is about	UUI-1	at t6	And about Type 2 diabetes mellitus
IUI-6	concretizes	IUI-8	at t6	It also concretizes the diagnosis
IUI-10	inheres in	IUI-9	at t9	The IQE inheres in the sentence on paper
IUI-10	is about	IUI-7	at t10	The IQE stands in aboutness to IUI-7
IUI-10	is about	IUI-1	at t10	It is also about Mr. Jones
IUI-10	is about	IUI-2	at t10	And about Mr. Jones’ disease
IUI-10	is about	UUI-1	at t10	And about Type 2 diabetes mellitus
IUI-10	concretizes	IUI-8	at t10	
IUI-10	is conformant to	IUI-6	at t10	Is conformant to the cognitive representation as long as it exists
IUI-3	agent in	IUI-11	at t11	
IUI-12	input into	IUI-11	at t15	Clinical picture input into IUI-11
IUI-6	output of	IUI-11	at t16	Cognitive representation output from IUI-11
IUI-8	output of	IUI-11	at t16	Both the diagnosis and its concretization are outputs of IUI-11
IUI-8	input into	IUI-13	at t17	The diagnosis is input into writing
IUI-6	input into	IUI-13	at t18	As is its cognitive representation
IUI-10	output of	IUI-13	at t19	The sentence is output of writing

**Table 7:** Relationships of representations to portions of reality in Scenario 3: *Incorrect diagnosis*

Relationships among particulars				Notes
IUI-46	is about	IUI-1	at t46	Dr. Jane Miller’s cognitive representation is about Mr. Jones
IUI-46	is about	IUI-2	at t46	And Mr. Jones’ disease
IUI-46	is about	UUI-2	at t46	And Type 1 diabetes mellitus (denoted by UUI-2)
IUI-50	is about	IUI-1	at t50	Likewise with the IQE inhering in the ink on paper
IUI-50	is about	IUI-2	at t50	
IUI-50	is about	UUI-2	at t50	
IUI-46	is misrepresentation of	IUI-7	at t46	But the cognitive representation is a misrepresentation of the configuration, i.e., it is intended to be about the configuration but fails on the level of compound expression
IUI-50	is misrepresentation of	IUI-7	at t50	The same is true of the IQE

To simplify our analysis somewhat, we wrote scenarios under which humans record diagnoses on paper. However, concretization of ICEs also occurs by pixels on monitors, binary switches in memory and processor chips, and magnetic fields on hard disks. But a detailed account of these concretizations and transformations among them is not central to our analysis of what is a diagnosis. Our analysis can be extended to these concretizations without modification of the method.

## Results and discussion

In each scenario, Mr. Jones (IUI-1) and his disease (IUI-2) exist, the latter inhering in the former (Table 3). Furthermore, his disease is an instance of the type ‘type 2 diabetes mellitus’ at any moment in time during which a diagnosis is formulated in any of the scenarios. Mr. Jones (IUI-1) exists through a certain period of time (*t1*) of which we do not know the exact beginning or end. We use temporal identifiers of the form ‘*t*n’ to clearly distinguish such identifiers from IUIs: where IUIs are always intended to be globally and singularly unique, distinct temporal identifiers may denote a unique period of time which is also denoted by another temporal identifier. We also assign an identifier to the time interval during which his disease (IUI-2) exists (*t2*). Diseases usually begin to exist after the organism does, but in the case of congenital genetic diseases, the two intervals might be coextensive. Also, we assume that disease IUI-2 existed at the time of diagnosing, but we recognize that diagnosing a disease thousands of years after it existed is possible, such as in the case of archaeologists’ recent diagnosis of Tutankhamun’s malaria [33].

Note that the configuration of organism, disease, and disease type is anchored at a particular location in spacetime, as is the diagnosis. But note also that the diagnosis additionally has an implicit or explicit reference to the location of the configuration in spacetime. To be a correct diagnosis, this reference must also be correct (it has to refer to some part, not necessarily the entirety of spacetime, occupied by the configuration). Thus, for example, to say that Tutankhamun had malaria in 1000 C.E. or today is incorrect, as it would be to say that Mr. Jones had type 2 diabetes mellitus before his parents were born.

### Scenario 1: correct diagnosis

In this scenario, numerous PORs in addition to Mr. Jones and his disease must exist and stand in certain relationships to each other (Tables 4, 5 and 6). Before Dr. Smith (IUI-3) writes (IUI-13) her diagnosis (IUI-8), there is a cognitive representation (IUI-6) that is concretized in some anatomical part (IUI-5) of her cognitive system (IUI-4). Note that we follow Ceusters and Smith [34] in asserting that all anatomical entities in which cognitive representations inhere are part of a person’s cognitive system (that is, any entity used in cognition, including the bearing of cognitive representations, are necessarily within a person’s cognitive system) at least during the temporal interval that the cognitive representation exists. If, for example, it would be the case that some white blood cell flowing through some brain capillary would through some of its molecules take part in the concretization of a cognitive representation, then that white blood cell would be part of the cognitive system at least during the existence of that concretization. It would not anymore be part of the cognitive system once it continues its journey through the body without participating in thought formation. Additionally, Ceusters and Smith take the position (which we also follow) that the cognitive system is not necessarily strictly limited to the brain or even to the entire neurological system of a person: the current state-of-the-art of neuroscience is yet searching for answers to questions such as “what is it in which cognitive representations inhere?” but until it reaches such answers, we remain in our representations agnostic.

IUI-9 denotes the sentence Dr. Smith wrote, as it exists on the particular piece of paper she used to write it on: ‘The patient has type 2 diabetes mellitus’. This written statement on paper (IUI-9) bears an information quality entity (IQE, IUI-10) that concretizes her diagnosis (IUI-8). The cognitive representation (IUI-6) and IQE (IUI-10) that concretize the diagnosis are both about the configuration (IUI-7) (the level of compound expression), as well as about Mr. Jones, Mr. Jones’ disease, and the universal *Type 2 diabetes mellitus* individually (the level of reference). The cognitive representation (IUI-6) and the diagnosis (IUI-8) are the output of Dr. Smith’s diagnostic process (IUI-11), which had as input Dr. Smith’s clinical picture (IUI-12) of Mr. Jones. Because the cognitive representation and IQE concretize the same ICE, the latter is conformant to the former (see Table 1).

**Fig. 2:**
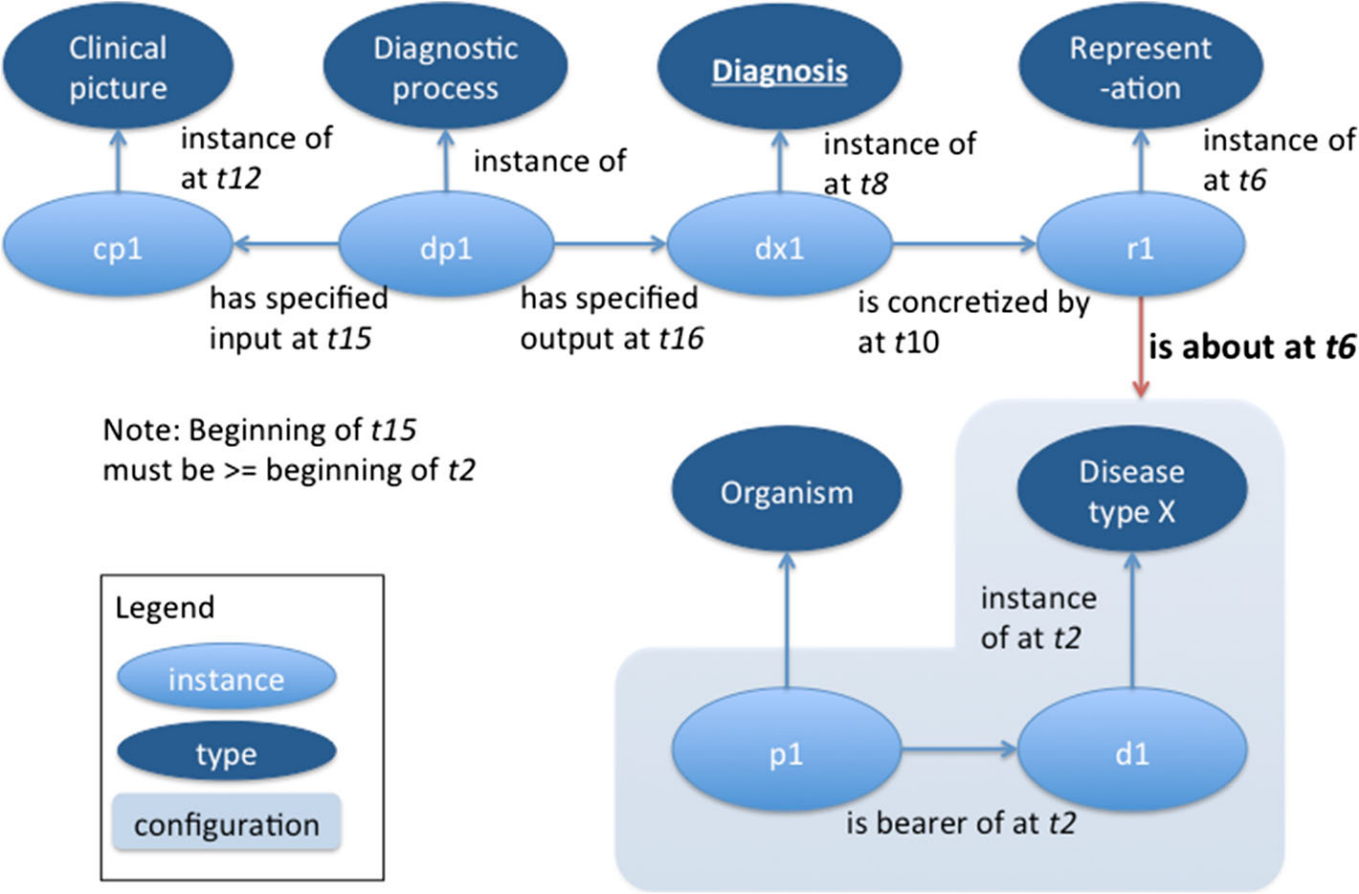
Diagram of diagnostic process, its inputs, a correct diagnosis, its concretization, and the configuration that that the concretization is about

*A correct diagnosis is thus fundamentally an information content entity that is concretized by a representation that stands in an is_about relation to the configuration of an organism, its disease, the relation of inherence between the disease and the organism, a type that the disease instantiates, and the instantiation relation of the disease to that type*, all within a given portion of spacetime (Fig. 2). Furthermore, diagnoses are additionally differentiated from other ICEs by the fact that they are generated by a diagnostic process that has a clinical picture as input. We expand further on what constitutes a clinical picture in the next scenario, *Scenario 2*, as well as revisit the diagnostic process briefly in *Scenario 4*, although it was not our objective in this work to develop a fuller account of this process.

Note that it is trivial to state that the particular disease inhering in the organism is an instance of *entity* or even *disease*. Thus, there is an expectation that a diagnosis be as precise (the most specific type) as possible and at a minimal level of granularity that is relevant to treat the patient appropriately and to provide a reasonable prognosis.

### Scenario 2: second diagnosis

The second physician, Dr. Brown, makes a second diagnosis at a later point in time, using the first diagnosis in addition to clinical and possibly other findings to infer a new clinical picture of Mr. Jones. With the exception of the configuration of Mr. Jones/his disease/type 2 diabetes mellitus (IUI-7), there is a one-to-one correspondence of PORs as in Scenario 1, numbered IUI-23 through IUI-33 (Additional file 1: Tables S1-S3). That is, there is no IUI-27 because the configuration is the same POR across scenarios. Similarly, there is no IUI-21 or IUI-22 because IUI-1 and IUI-2 already identify Mr. Jones and his disease, respectively, uniquely.

In this scenario, Dr. Brown (IUI-23) makes a new diagnosis (IUI-28), concretized both by his cognitive representation (IUI-26) in some part (IUI-25) of his cognitive system (IUI-24) and by the IQE (IUI-30) inhering in the sentence in his note (IUI-29). Dr. Smith’s previous diagnosis (IUI-8) can be viewed as either (*view1*) being in the aggregate of things that Dr. Brown uses to infer his clinical picture (IUI-32) that serves as input into his diagnostic process (IUI-31), or (*view2*) as something which serves as extra input—alongside his clinical picture—for the diagnostic process. The cognitive representation and the IQE are about the configuration (IUI-7) as well as Mr. Jones (IUI-1), his disease (IUI-2), and type 2 diabetes mellitus (UUI-1).

The current definition of ‘clinical picture’ in OGMS (see Table 2) seems to conflict with *view1* about this scenario, because the definition seems to exclude using a past diagnosis to infer a clinical picture. Although the current OGMS definition of ‘clinical picture’ is inclusive of clinical findings, diagnosis as currently defined is not an explicit subtype of clinical finding in OGMS. Furthermore, it is common for clinicians to elicit a previous provider’s past diagnosis from the patient or the patient’s caregiver during an interview (for example, if Mr. Jones in scenario #2 would have said: ‘Dr. Smith says I have type 2 diabetes mellitus’). But the current OGMS definition of ‘clinical history’ (Table 2) conflicts with this possibility. It refers to health-relevant features of a patient, but features as elucidated by OGMS include only qualities, processes, and physical components of the organism—not dispositions of which disease is a subtype. Therefore, a representation of a disease such as a diagnosis is currently excluded from the OGMS definition of ‘clinical history’.

We also note that the OGMS definition of ‘clinical picture’ is ambiguous in that it is not clear whether it *requires* that laboratory and image findings must always be used to infer a clinical picture, or that they are the only entities that can be used. Regardless, it would be a mistake to do so, because diagnoses can and frequently are made from symptom findings alone. Laboratory and image findings are not necessary components of a clinical picture in reality. Note that a clinical picture can comprise findings of a single type (laboratory alone, pathology image alone, radiology image alone, physical exam finding alone), or even a single finding instance (e.g. Reed-Sternberg cells for a diagnosis of Hodgkin’s lymphoma). All these issues are compounded by the fact that the term ‘clinical picture’ itself is not intuitive.

Given that clinical history taking elicits past diagnoses routinely in clinical medicine, we propose modifying the definition of ‘clinical history’ to accommodate this reality (bolded sections represent changes to the definition):

**clinical history = def.** – *A series of statements representing one or more health-relevant features of a patient****, possibly complemented by representations of diseases and configurations****.*

Note that the definition already allows—under the broader heading of ‘feature’—representations of disorders (kinds of physical component) and disease courses (kinds of process). Thus, the definition already accommodates these aspects of clinical histories. We also allow the statements to represent configurations, in line with Smith and Ceusters [2]. These configurations might or might not include various relevant types (for example, “The patient has not participated in any instance of vomiting in the last two weeks.”). Finally, note that by using the word ‘representing’, the definition also accommodates per Smith and Ceusters [2] that some statements might fail in aboutness despite their intention to be about such features. In other words, some statements in the clinical picture might be wrong: for example, a statement that the patient has a disease or pain that she does not in fact have.

To clarify that laboratory and imaging findings are not always required inputs into the diagnostic process, and to capture realistic scenarios compatible with *view2* (for example, Dr. Brown reads Dr. Smith’s note in the chart), we also propose a modified definition of ‘clinical picture’ (changes in bold):

**clinical picture = def.** – *A representation of a clinical phenotype that is inferred from****a****combination of,****for example, diagnoses and****laboratory, image, and clinical findings about a given patient.*

These changes to the definitions of ‘clinical history’ and ‘clinical picture’ now properly capture situations where past diagnoses are elicited from the patient and/or her caregiver during a clinical history taking: these diagnoses are now clinical findings in the clinical history that was generated by the clinical history taking (see the definition of ‘clinical finding’ in Table 2).

### Scenario 3: Misdiagnosis

**Fig. 3:**
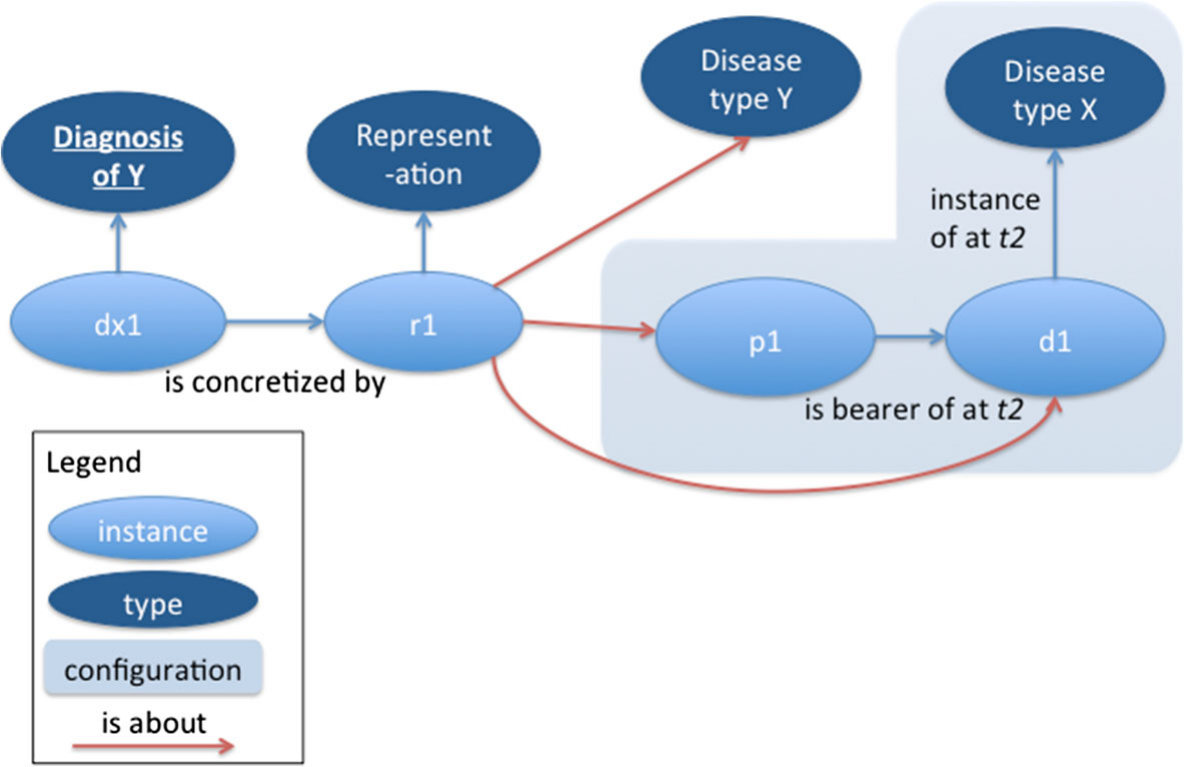
Misdiagnosis of type of disease. The diagnosis is individually about the patient, the disease, and the incorrectly diagnosed disease type Y, but it is not about the configuration of patient, disease, and disease type X

The third physician, Dr. Miller, misdiagnoses Mr. Jones’ type 2 diabetes mellitus as type 1 diabetes mellitus (Fig. 3). Per Smith and Ceusters, because the misdiagnosis is still about Mr. Jones, his disease, the relationship between them, and the type ‘type 1 diabetes mellitus’ on the level of reference, it is an information content entity. However, it fails to be about the configuration IUI-7 as a whole on the level of compound expression.

Again, in this scenario there exist PORs in one-to-one correspondence (except the configuration and its components) numbered IUI-43 through IUI-53 (Additional file 2: Tables S4-S6). Dr. Miller (IUI-43) writes (IUI-53) his misdiagnosis (IUI-48) in Mr. Jones’ chart, and the IQE (IUI-50) inhering in the ink (IUI-49) is conformant to his cognitive representation (IUI-46), and both are about—on the level of reference—Mr. Jones, his disease, and type 1 diabetes mellitus. But neither one is about the configuration (IUI-7). To capture the relation both (1) between the cognitive representation and the configuration and (2) between the IQE and the configuration, we define a new relation:

**is-misrepresentation-of**: domain: representation, range: portion of reality.

Def: *x* is-misrepresentation of *y* iif *x* is a representation and *x* is intended to be about *y* and it is not the case that *x* is about *y*.

Then we assert that the representations (IUI-46 and IUI-50) are misrepresentations of the configuration (Table 7 and Additional file 2: Table S6). Note that our definition precludes the cognitive representation (IUI-46) and IQE (IUI-50) being about any configuration other than IUI-7, because they are not intended to be about, for example, the configuration of the sun, earth, and moon at a particular date and time.

Note that asserting the incorrect disease type is not the only way to make a misdiagnosis. There are at least six possibilities where a diagnosis fails to be about a configuration on the level of compound expression (Table 8). If a representation fails on the level of reference, it also fails on the level of compound expressions, because a configuration cannot consist of that which does not exist. These six possibilities could also exist in combination, but if the 2nd, 3rd, and 4th possibilities are all present (for example, “Ron Weasley has spattergroit”), then there is not a diagnosis, or even any information content entity at all, because the representation is not about anything even on the level of reference. Of course, if the organism itself does not exist, then there cannot be a clinical picture inferred, and thus it would not be a diagnosis or misdiagnosis, although it could still be an ICE if it is about a really-existing disease type (for example, “James Bond has influenza”). Also, as medical knowledge evolves, the profession comes to understand that certain types of disease thought to exist in fact do not. Thus past diagnoses of *dropsy* and *consumption* we now understand to be misdiagnoses.

**Table 8:** Six possibilities for a diagnosis failing in aboutness on the level of compound expressions

Problem	Where it fails *first*	Description
Noninstantation, asserted type exists	Level of compound expression	Disease instantiates a different type than the stated type, but the stated type exists
Noninstantation, asserted type does not exist	Level of reference	Disease instantiates a different type than stated, while the stated type of disease does not exist
Disease nonexistence	Level of reference	The disease instance does not exist
Organism nonexistence	Level of reference	The organism instance does not exist. In this case, there could not be a clinical picture properly inferred and thus it is not a misdiagnosis although it could still be an ICE.
Disease non-inherence	Level of compound expression	The disease inheres in a different organism than the one stated. For example, the doctor mistakenly ascribes Mr. Johnson’s hypertension to his twin.
Configuration is not located in that part of spacetime where the diagnosis says it is located.	Level of compound expression	A diagnosis of type 2 diabetes mellitus 5 years ago is wrong because the patient didn’t have the disease at that time, even though the patient has type 2 diabetes today. Also, a diagnosis that the patient has an upper respiratory tract infection today when in reality the infection resolved two weeks ago.

Despite searching the extensive literature on diagnostic error, we could not find any studies that looked at what percentages of misdiagnoses fall into these categories. We conjecture based on our past clinical expertise and experience that asserting the incorrect disease type is the most common mistake among those in Table 8, but confirmation or rejection of this conjecture requires study.

### Scenario 4: the lucky guess

In this scenario, a layperson (the “seer”—IUI 63) correctly concluded coincidentally that Mr. Jones had type 2 diabetes mellitus based on the position of the moon and Mr. Jones’ horoscope (Additional file 3: Tables S7-S9). It would be wrong to say the seer’s reasoning (IUI-71) constituted a diagnostic process. To avoid coincidentally correct statements from qualifying as diagnoses, we additionally require as input into the diagnostic process cognitive representations of the disease type and the types instantiated by the sequalae, signs, symptoms, and any clinical, laboratory, or imaging findings or phenotypes of the instances of this disease type. Note that this is a minimal requirement: clinicians often additionally include in their diagnostic reasoning cognitive representations of other disease types and associated PORs when considering alternative possibilities for the disease type.

This view is based on the extensive literature on clinical reasoning processes, especially diagnosis (for a review, see Norman [35]). This research has established the use of representations, called ‘knowledge structures’, in the diagnostic process. The nature and form of these representations evolves as clinical expertise develops [36], and we note that the differences in diagnostic processes that result could result in a typology of diagnostic processes in OGMS.

Because the seer had no cognitive representations of type 2 diabetes mellitus, let alone used them as input into his “reasoning”, his conclusion (IUI-68), although an ICE, is not a diagnosis. Similarly, if a physician makes a lucky guess based not on his cognitive representations of the stated disease type but instead by flipping a coin or some such, that too would not be a diagnosis.

To Table 3 we add an aggregate of cognitive representations of disease types and associated entities as input into the diagnostic process (Table 9).

**Table 9:** Additional tuples required to distinguish diagnosing from a lucky guess

IUI	Entity	Lifetime	Type	Notes
IUI-14	The aggregate of Dr. Smith’s cognitive representations of various disease types and their associated types of phenotypes including type 2 diabetes mellitus that he used in the diagnostic process	t20	Aggregate of cognitive representations	
Relationships among particulars				
IUI-14	input into	IUI-11	at t21	t21 refers to the temporal interval during which IUI-14 participated in the reasoning process. It could start at the same time as t11 or after t11, and end at the same time as or before t11.

We propose to redefine diagnostic process as follows:

**Diagnostic process = def**. *An interpretive PROCESS that has as input (1) a CLINICAL PICTURE of a given patient****AND (2) an aggregate of REPRESENTATIONs of at least one type of disease and at least one type of phenotype whose instances are associated with instances of that disease,****and as output an assertion to the effect that the patient has a DISEASE of a certain type.*

### Scenario 5: layperson’s justifiable conclusion

Mr. Jones’ daughter wrote a sentence in her letter to her brother based on reading Dr. Smith’s progress note saying that that her father has type 2 diabetes mellitus (Additional file 4: Tables S10-S12). Of course, the daughter has not made a diagnosis. She is communicating to her brother what she believes to be the case.

Had she merely written “Dr. Smith says” and then copied Dr. Smith’s sentence word for word into her letter, then her writing would concretize Dr. Smith’s diagnosis (IUI-8). This is the case of hearsay (“so-and-so said it was the case that…”).

As Smith and Ceusters showed, however, the same sentence written by two different people does not guarantee they concretize the same ICE. ICEs are further differentiated by the provenance of their concretizations, including who created them and when, and to what POR they intend to be about. In their example, two people writing the sentence *Barack Obama has never been President of the United States*—one before and one after Obama’s inauguration as President—generate two different ICEs. The one written after fails on the level of compound expressions but not on the level of reference, whereas the one written before succeeds on both levels (it remains true that at the time when the sentence was written, he had never been President).

We therefore distinguish between a human (1) merely copying a representation, in which case the copy concretizes the same ICE as the original text and (2) creating her own cognitive representation of the POR—which involves forming a belief that the POR really existed as represented—and then subsequently creating an IQE that is conformant to the cognitive representation. In the former case, a new ICE does not come into being. It does not even require in the cognitive system of the copier any representation of the POR that the original representation is about (as in the case of copying German text that one does not understand at all). In the latter case, by contrast, a new ICE does come into being.

In Scenario 5, the daughter did not merely repeat Dr. Smith’s diagnosis. She communicated to her brother *her* belief about her father’s disease. She deliberately chose not to merely convey Dr. Smith’s diagnosis, but rather her belief that her father has type 2 diabetes mellitus. She heard the opinion of an expert, in whom she had trust. Based on (1) her observations of her father, (2) Dr. Smith’s diagnosis, and (3) her trust in Dr. Smith, she reached the conclusion herself that her father suffers from type 2 diabetes mellitus. Because she did not begin with a clinical picture and her own cognitive representations of type 2 diabetes mellitus, her conclusion is not a diagnosis.

However, consider the scenario where she is given the clinical picture and has enough knowledge to arrive at a conclusion, which could be the case either if she were a physician or somehow other acquired or were given the necessary knowledge: it is analagous to Scenario #6, where she takes the place of the expert system (see analysis of that scenario below). Thus, here in Scenario #5 it is important to note that she did not reason from a clinical picture to the diagnosis.

In this scenario, therefore, the daughter has created a new ICE (IUI-88) that is not a diagnosis. She has concretized it in the sentence (IUI-89) in her letter.

### Scenario 6: diagnosis by non-human

The diagnostic decision support system has made a diagnosis (or misdiagnosis depending on whether it is correct), because it (1) takes as input a clinical picture and representations of the relevant disease type and one or more types of phenotypes with which it is associated; (2) participates in a process of making a conclusion based on this input; and (3) outputs from this process a statement about a configuration involving an organism, a disease, and a disease type.

In this case, there are no cognitive representations. In their place are digital representations on hard drives, memory chips, and central processing units. If we assume the system generates a sentence and prints it on paper, then we have an analagous IQE to the written diagnosis of the physician and ICE of the sister.

Nothing in our proposed definitions conflicts with this scenario. Replacing Dr. Smith and associated representations and diagnostic process with various components of the computer and its digital representations as well as inferential process (which is an instance of diagnostic process) is straightforward.

Returning briefly to a point made in Scenario #5, Mr. Jones’ daughter could follow the exact same algorithm(s) of the diagnostic expert system using the exact same clinical picture as input, and she would arrive at (or make) a diagnosis, in contrast to scenario #5 where her conclusion was an ICE but not a diagnosis.

## Conclusions

We applied Smith and Ceusters’ results on aboutness [25] to diagnosis in order to develop an account of diagnosis, misdiagnosis, lucky guesses, hearsay, a layperson’s justified belief about disease configurations, and a diagnosis made by an expert system. Our key result is that a correct diagnosis, as defined by OGMS, is about a configuration of an organism, its disease, and the type the disease instantiates (level of compound expression) in a specified portion of spacetime. A misdiagnosis by contrast is a misrepresentation of this configuration. Nevertheless, both diagnosis and misdiagnosis are still about—at the level of individual reference—the organism and (when they exist) a disease instance and a disease type. Also, they are both the output of a diagnostic process, which differentiates them from lucky guess and hearsay as well as the misinformation-based counterparts to lucky guess and hearsay. We also carefully represented the inputs and outputs of this process.

We identified several subtypes of misdiagnosis (e.g., wrong disease subtype, wrong patient, wrong temporal placement) that have not been differentiated in the literature on diagnostic error, to our knowledge. Studying the incidence and causes of these subtypes might advance the study of diagnostic error and strategies to reduce it. Note that as we have defined it, ‘misdiagnosis’ does not refer to the diagnostic errors of absent diagnosis (failing to diagnose a disease at all, let alone incorrectly) and delayed diagnosis. Lastly, we note that the current literature on diagnostic error, per a 2016 Institute of Medicine report, does not lend itself to generating reliable estimates of incidence of diagnostic error per se, let alone any subtype of such error [37].

Although misdiagnoses involving non-existence of certain entities might at first seem to be of minor importance, we highlight two cases where non-existence is relevant. First, in the case where the type of disease does not exist (consider past diagnoses of “dropsy”), it could well be that our understanding of disease decades from now is much more advanced, and what we think are types of disease today in fact are not. So just as with past diagnoses of “dropsy”, it could be that today’s diagnoses of “schizophrenia” are misdiagnoses merely by referring to a type that does not exist. Second, in the case where the instance of disease does not exist, we consider two scenarios. The first scenario involves past diagnoses of mental illness where neither the instance nor the type exists. For example, past diagnoses of runaway slaves as having “drapetomania” involved neither a really existing instance nor a really existing type of disease. The second scenario involves patients with hypochondria or who are malingering. They feign a condition for which the unassuming practitioner mistakenly asserts the existence of an instance and the instantiation of a type.

Our results and typology of misdiagnosis could serve as the beginnings of a formal framework for studying diagnostic error as a component of data quality in EHRs and research data collections, in response to the call by Weiskopf and Weng for more formal, generalizable, and validated methods for assessing data quality [24]. Applying Ceusters’ detailed typology of mistakes in ontology (e.g., asserting a type that does not exist) [38] and referent tracking systems (e.g., assigning an identifier but there is no corresponding particular that it identifies, assigning one identifier to two particulars, assigning two identifiers to one particular, etc.) [39] to diagnosis could build on our work here to build out such a framework. It remains future work to do so.

The provenance of the ICE and its concretizations are critical: lucky guesses, hearsay, and laypersons’ conclusions about disease (when not arrived at through a diagnostic process using a clinical picture and cognitive representations of the associated type(s) of disease as input) do not constitute diagnoses and therefore are different types of ICE than diagnoses. Provenance also includes which findings and other information constituted the clinical picture used in the diagnostic process. Our analysis of the scenarios identified past diagnoses as important input into the diagnostic process, leading to proposed redefinitions of ‘clinical history’, ‘clinical picture’, and ‘diagnostic process’ for OGMS.

Smith and Ceusters’ results on aboutness and our extension of them here to diagnosis reduce the need for the workarounds reported by Martínez Costa and Schulz [26] and Hastings et al. [27] It is perfectly legitimate to relate ‘suspected heart failure finding’ to ‘congestive heart failure’ with an existential quantifier: if an instance of this type is not about a really-existing configuration of patient–disease–heart failure, it is still an ICE that is individually about the patient, her condition, and the type *heart failure* on the level of reference. In OWL, we could assert:

- *Suspected heart failure ICE* - > ICE and (**is about** SOME *Organism*)
- *Suspected heart failure ICE* - > ICE and (**is about** SOME *Condition*)

In more expressive formalisms including first-order logic, we could also assert that it is about the type *heart failure*, where ‘Type’, ‘Instance_of’, and ‘Is_about’ are predicates in what follows, where the universal quantification applies to the ICE, not what it is about:

- Type(heart_failure)
- Type(suspected_heart_failure_ICE)
- ∀*x* (Instance_of(*x*, suspected_heart_failure_ICE) - > Is_about(*x*, heart_failure))

Similarly, chemical graphs and diagrams are ICEs about individual types of atoms such as carbon, oxygen, hydrogen, and so on, even when they fail to be about any type of configuration (e.g., molecule) of such atoms. However, because they are typically not about any instances, proper existential quantification in OWL is not possible. However, we could relate in first-order logic the diagram of *octaazacubane* (a hypothetical molecule which would be comprised of eight nitrogen atoms arranged in a cubic structure) to the *nitrogen* type of atom using existential quantification (again where the universal quantification in what follows applies to the ICE and not what it is about):

- Type(nitrogen_atom)
- Type(octaazacubane _diagram)
- ∀*x* (Instance_of(*x*, octaazacubane_diagram) - > Is_about(*x*, nitrogen_atom))

It is therefore not required to use universal quantification over the range of things that an ICE is about, when relating ICEs to those entities they are about, to avoid failure of aboutness on the level of compound expression. This result is qualified by the constraints of representational formalisms such as OWL that prevent directly asserting aboutness to types. Schulz et al. describe workarounds in OWL to asserting aboutness to types, that may be of benefit in some use cases [40].

The use of universal quantification actually introduces problems when we account for aboutness on the level of individual reference. For example, if we leave the ‘suspected heart failure finding’ of Martínez Costa and Schulz as being *only* about ‘congestive heart failure’, then it would result in a contradiction to say that it is about some organism. Likewise for condition. So use of the universal quantifier precludes aboutness on the level of individual reference, in direct conflict with the results of Smith and Ceusters on misinformation.

Although it was not the primary or even secondary goal of the present work, other advantages of our approach with respect to inference are easy to derive. First, in our approach with explicit representation of the disease in addition to the diagnosis, we can infer all instances of Type 1 diabetes mellitus that have been misdiagnosed as Type 2 diabetes mellitus at some point in time, in first order logic minimally and possibly in OWL with workarounds. Generalizing slightly, we can query for all conditions that have been misdiagnosed as Type 2 diabetes mellitus. Using a typology of organisms, we can find in the veterinary domain all diagnoses and/or misdiagnoses of a certain type of disease in organisms of a certain type: for example, misdiagnoses of foot and mouth disease in cattle. Having no ability to create an aboutness relation from a misdiagnosis, or more generally an incorrect clinical statement, to the organism it is about (due to the contradictions that will result as pointed out above) or even to anything in reality at all, the universal quantifier approach of Martínez Costa and Schulz would require substantial revision to make these inferences.

In the realm of chemical diagrams, our approach enables one to query for all chemical diagrams that depict nitrogen atoms or certain chemical groups (e.g., hydroxyl group and benzene rings), *including the diagrams that are not about any existing type of molecule*. The universal quantifier approach in Hastings et al., by contrast, would require significant revision to return diagrams that depict nitrogen, hydroxyl groups, benzene rings, and so on, but are not about any existing type of molecule. In depth exploration of the effects of our representation on inference remains future work, as it is not our primary interest here.

Our analysis also identified problems with, and suggested improvements to, the definitions of core terms from the Ontology for General Medical Science including ‘diagnostic process’ and ‘clinical picture’. This result is consistent with our past work, where we have found the method of referent tracking analysis to be a stringent test of definitions in ontologies.

This work is limited by the fact that we did not conduct further ontological analysis of the diagnostic process beyond OGMS and beyond what our scenarios required, as this was not the purpose of the present work. We do note that our requirement for including cognitive representations of disease types as input into the diagnostic process is based on this literature, however. Engaging experts in the study of clinical reasoning in future work to develop a typology of diagnostic processes has the potential to result in a corresponding typology of diagnoses.

Future work includes (1) an account of differential diagnosis, where a clinician or expert system generates a list of likely types of disease for further investigation to identify the actual type the organism’s disease instantiates; (2) proposing to the OGMS community to clarify the definitions of ‘clinical history’, ‘clinical picture’, and ‘diagnostic process’ as suggested here, and to expand the definition of diagnosis to include disorders, disease courses, and absence of disease (i.e., healthy); (3) extending our analysis as reported here to this expanded definition of ‘diagnosis’; (4) conducting deeper ontological analysis of the diagnostic process, in coordination with experts in the study of clinical reasoning; and (5) more fully exploring the effects of our representations on logical inference beyond some readily evident advantages discussed here.

## Additional files

Additional file 1: Table S1. Entities in Scenario 2: *Second correct diagnosis.* **Table S2**. Additional temporal entities in Scenario 2: *Second correct diagnosis*. **Table S3**. Relationships among particulars in Scenario 2: *Second correct diagnosis*. (DOCX 88 kb) Additional file 2: Table S4. Entities in Scenario 3: *Incorrect diagnosis.* **Table S5**. Additional temporal entities in Scenario 3: *Incorrect diagnosis.* **Table S6**. Relationships among particulars in Scenario 3: *Incorrect diagnosis. (DOCX 88 kb)*
Additional file 3: Table S7. Entities in Scenario 4: *Layperson’s unjustified inference*. **Table S8**. Additional temporal entities in Scenario 4: *Layperson’s unjustified inference*. **Table S9**. Relationships among particulars in Scenario 4: *Layperson’s unjustified inference*. (DOCX 95 kb) Additional file 4: Table S10. Entities in Scenario 5: *Layperson’s justified inference.* **Table S11.** Additional temporal entities in Scenario 5: *Layperson’s justified inference.* **Table S12**. Relationships among particulars in Scenario 5: *Layperson’s justified inference. (DOCX 92 kb)*

## Abbreviations

BFO: Basic formal ontology
GDC: Generically dependent continuant
IAO: Information artifact ontology
ICE: Information content entity
IQE: Information quality entity
OGMS: Ontology for General Medical Science
POR: Portion of reality
RT: Referent tracking
RTT: Referent tracking tuple
