# Supplementary material for: Diagnosis, misdiagnosis, lucky guess, hearsay, and more: an ontological analysis
Source: J Biomed Semantics. 2016 Sep 15;7:54. doi: 10.1186/s13326-016-0098-5 (PMC5025551; doi:10.1186/s13326-016-0098-5)
Supplement: Additional file 1: Table S1. — Entities in Scenario 2: Second correct diagnosis. Table S2. Additional temporal entities in Scenario 2: Second correct diagnosis. Table S3. Relationships among particulars in Scenario 2: Second correct diagnosis. (DOCX 88 kb) [file 13326_2016_98_MOESM1_ESM.docx]

**Table S1**. Entities in Scenario 2: *Second correct diagnosis*

| **IUI** | **Entity** | **Existence period** | **Type** | **Notes** |
| --- | --- | --- | --- | --- |
| IUI-23 | Dr. John Brown | t23 | Human being |  |
| IUI-24 | Cognitive system of IUI-23 | t24 |  |  |
| IUI-25 | An anatomical entity that is part of IUI-24 | t25 | Anatomical entity | Which anatomical entity and its lifetime cannot be easily specified given current state of neuroscience. |
| IUI-26 | Quality that inheres in IUI-25 and is about IUI-7 | t26 | Cognitive representation |  |
| IUI-7 | The POR that is truth-maker for IUI-28 | t7 | Configuration | Mr. Jones, his disease, their relationship, and disease’s instantiation |
| IUI-28 | Dr. Brown’s diagnosis | t28 | Diagnosis | ICE concretized by IUI-26 & IUI-30 |
| IUI-29 | That which is written down on paper and forms the sentence. | t29 | Material entity | *I conclude therefore that Mr. Jones has type 2 diabetes mellitus.* |
| IUI-30 | IQE that inheres in IUI-29. | t30 | Information quality entity | The sentence began to exist as soon as ink was laid down on paper, but the IQE did not begin to exist until the sentence was finished. |
| IUI-31 | Dr. Brown’s interpretive process | occupies t31 | Diagnostic process | Dr. Brown’s interpretive process that led to her diagnosis IUI-28 |
| IUI-32 | The clinical picture input into IUI-31 | t32 | Clinical picture | Dr. Brown’s clinical picture as ascertained prior to t25 |
| IUI-33 | Dr. Brown writing his diagnosis in the note | occupies t33 | Process |  |

**Table S2.** Additional temporal entities in Scenario 2: *Second correct diagnosis*.

| **Temporal identifier** | **Description** | **Notes** |
| --- | --- | --- |
| t34 | The interval during which the anatomical entity (IUI-25) is part of the cognitive system (IUI-24) | This interval is not easily specified given the current state of neuroscience. It could be different than t23 and t24. |
| t35 | The interval during which the clinical picture (IUI-32) is used in the interpretive process (IUI-31) | Could be shorter than t31 |
| t36 | The point in time at which the cognitive representation (IUI-26) and diagnosis (IUI-28) begin to exist | t36 ends t31. Because the ICE does not exist until the cognitive representation—its first concretization—exists, this is also the point in time at which the diagnosis begins to exist. |
| t37 | The interval during which the cognitive representation (IUI-26) participates in the writing process (IUI-33) |  |
| t38 | The interval during which the diagnosis (IUI-28) participates in the writing process (IUI-33) | It is possible that the original cognitive representation (IUI-26) gets copied elsewhere in the brain for reasoning and thus that the ICE continues to participate after the initial cognitive representation |
| t39 | The interval during which that which is written on paper (IUI-30) begins to exist until it exists in full | The writing process begins earlier than the time at which the sentence begins to exist: the author starts the process with getting a pen and paper, any preparation necessary (“clicking” the pen), etc. |

**Table S3.** Relationships among particulars in Scenario 2: *Second correct diagnosis*.

| **IUI** | **Relation** | **IUI** | **When relation holds in reality** | **Notes** |
| --- | --- | --- | --- | --- |
| IUI-24 | **part of** | IUI-23 | at t24 |  |
| IUI-25 | **part of** | IUI-24 | at t25 | All anatomical components in which the cognitive representation inheres are part of the cognitive system. We do not assume the cognitive system is limited to the brain or even nervous system. |
| IUI-26 | **inheres in** | IUI-25 | at t26 |  |
| IUI-26 | **is about** | IUI-7 | at t26 | The cognitive representation stands in aboutness to IUI-7 as long as it exists |
| IUI-26 | **is about** | IUI-1 | at t26 | It is also about Mr. Jones |
| IUI-26 | **is about** | IUI-2 | at t26 | And about Mr. Jones’ disease |
| IUI-26 | **is about** | UUI-1 | at t26 | And about Type 2 diabetes mellitus |
| IUI-26 | **concretizes** | IUI-28 | at t26 | It also concretizes the diagnosis |
| IUI-30 | **inheres in** | IUI-29 | at t29 | The IQE inheres in the sentence on paper |
| IUI-30 | **is about** | IUI-7 | at t30 | The IQE stands in aboutness to IUI-7 |
| IUI-30 | **is about** | IUI-1 | at t30 | It is also about Mr. Jones |
| IUI-30 | **is about** | IUI-2 | at t30 | And about Mr. Jones’ disease |
| IUI-30 | **is about** | UUI-1 | at t30 | And about Type 2 diabetes mellitus |
| IUI-30 | **concretizes** | IUI-28 | at t30 |  |
| IUI-30 | **is conformant to** | IUI-26 | at t30 | The IQE is conformant to the cognitive representation as long as it exists |
| IUI-23 | **agent in** | IUI-31 | at t31 |  |
| IUI-32 | **input into** | IUI-31 | at t35 | Clinical picture input into IUI-31 |
| IUI-26 | **output of** | IUI-31 | at t36 | Cognitive representation output from IUI-31 |
| IUI-28 | **output of** | IUI-31 | at t36 | Both the diagnosis and its concretization are outputs of IUI-31 |
| IUI-28 | **input into** | IUI-33 | at t37 | The diagnosis is input into the writing process |
| IUI-26 | **input into** | IUI-33 | at t38 | As is the cognitive representation |
| IUI-30 | **output of** | IUI-33 | at t39 | The sentence is output of the writing process |
