# Supplementary material for: Diagnosis, misdiagnosis, lucky guess, hearsay, and more: an ontological analysis
Source: J Biomed Semantics. 2016 Sep 15;7:54. doi: 10.1186/s13326-016-0098-5 (PMC5025551; doi:10.1186/s13326-016-0098-5)
Supplement: Additional file 2: Table S4. — Entities in Scenario 3: Incorrect diagnosis. Table S5. Additional temporal entities in Scenario 3: Incorrect diagnosis. Table S6. Relationships among particulars in Scenario 3: Incorrect diagnosis. (DOCX 88 kb) [file 13326_2016_98_MOESM2_ESM.docx]

**Table S4**. Entities in Scenario 3: *Incorrect diagnosis*

| **IUI** | **Entity** | **Existence period** | **Type** | **Notes** |
| --- | --- | --- | --- | --- |
| IUI-43 | Dr. Jane Miller | t43 | Human being |  |
| IUI-44 | Cognitive system of IUI-43 | t44 |  |  |
| IUI-45 | An anatomical entity that is part of IUI-44 | t45 | Anatomical entity | Which anatomical entity and its lifetime cannot be easily specified given current state of neuroscience. |
| IUI-46 | Quality that inheres in IUI-45 and is about IUI-7 | t46 | Cognitive representation |  |
| IUI-7 | The POR that is truth-maker for IUI-48 | t7 | Configuration | Mr. Jones, his disease, their relationship, and disease’s instantiation |
| IUI-48 | Dr. Miller’s misdiagnosis | t48 | Diagnosis | ICE concretized by IUI-46 & IUI-50 |
| IUI-49 | That which is written down on paper and forms the sentence. | t49 | Material entity | *I conclude therefore that Mr. Jones has type 1 diabetes mellitus.* |
| IUI-50 | IQE that inheres in IUI-49. | t50 | Information quality entity | The sentence began to exist as soon as ink was laid down on paper, but the IQE did not begin to exist until the sentence was finished. |
| IUI-51 | Dr. Miller’s interpretive process | occupies t51 | Diagnostic process | Dr. Miller’s interpretive process that led to her misdiagnosis IUI-48 |
| IUI-52 | The clinical picture input into IUI-51 | t52 | Clinical picture | Dr. Miller’s clinical picture as ascertained prior to t45 |
| IUI-53 | Dr. Miller writing her misdiagnosis in the note | occupies t53 | Process |  |

**Table S5.** Additional temporal entities in Scenario 3: *Incorrect diagnosis.*

| **Temporal identifier** | **Description** | **Notes** |
| --- | --- | --- |
| t54 | The interval during which the anatomical entity (IUI-45) is part of the cognitive system (IUI-44) | This interval is not easily specified given the current state of neuroscience. It could be different than t43 and t44. |
| t55 | The interval during which the clinical picture (IUI-52) is used in the interpretive process (IUI-51) | Could be shorter than t51 |
| t56 | The point in time at which the cognitive representation (IUI-46) and misdiagnosis (IUI-48) begin to exist | t56 ends t51. Because the ICE does not exist until the cognitive representation—its first concretization—exists, this is also the point in time at which the misdiagnosis begins to exist. |
| t57 | The interval during which the cognitive representation (IUI-46) participates in the writing process (IUI-53) |  |
| t58 | The interval during which the misdiagnosis (IUI-48) participates in the writing process (IUI-53) | It is possible that the original cognitive representation (IUI-46) gets copied elsewhere in the brain for reasoning and thus that the ICE continues to participate after the initial cognitive representation |
| t59 | The interval during which that which is written on paper (IUI-50) begins to exist until it exists in full | The writing process begins earlier than the time at which the sentence begins to exist: the author starts the process with getting a pen and paper, any preparation necessary (“clicking” the pen), etc. |

**Table S6.** Relationships among particulars in Scenario 3: *Incorrect diagnosis.*

| **IUI** | **Relation** | **IUI** | **When relation holds in reality** | **Notes** |
| --- | --- | --- | --- | --- |
| IUI-44 | **part of** | IUI-43 | at t44 |  |
| IUI-45 | **part of** | IUI-44 | at t45 | All anatomical components in which the cognitive representation inheres are part of the cognitive system. We do not assume the cognitive system is limited to the brain or even nervous system. |
| IUI-46 | **inheres in** | IUI-45 | at t46 |  |
| IUI-46 | **is misrepresentation of** | IUI-7 | at t46 | The cognitive representation is intended to be about, but fails to be about, IUI-7 as long as it exists, so it is a misrepresentation of IUI-7 |
| IUI-46 | **is about** | IUI-1 | at t46 | But it is nevertheless still about Mr. Jones |
| IUI-46 | **is about** | IUI-2 | at t46 | And about Mr. Jones’ disease |
| IUI-46 | **is about** | UUI-2 | at t46 | And about Type 1 diabetes mellitus |
| IUI-46 | **concretizes** | IUI-48 | at t46 | It also concretizes the misdiagnosis |
| IUI-50 | **inheres in** | IUI-49 | at t49 | The IQE inheres in the sentence on paper |
| IUI-50 | **is misrepresentation of** | IUI-7 | at t50 | The IQE also misrepresents IUI-7 |
| IUI-50 | **is about** | IUI-1 | at t50 | But it is about Mr. Jones |
| IUI-50 | **is about** | IUI-2 | at t50 | And about Mr. Jones’ disease |
| IUI-50 | **is about** | UUI-2 | at t50 | And about Type 1 diabetes mellitus |
| IUI-50 | **concretizes** | IUI-48 | at t50 |  |
| IUI-50 | **is conformant to** | IUI-46 | at t50 | The IQE is conformant to the cognitive representation as long as it exists |
| IUI-43 | **agent in** | IUI-51 | at t51 |  |
| IUI-52 | **input into** | IUI-51 | at t55 | Clinical picture input into IUI-51 |
| IUI-46 | **output of** | IUI-51 | at t56 | Cognitive representation output from IUI-51 |
| IUI-48 | **output of** | IUI-51 | at t56 | Both the misdiagnosis and its concretization are outputs of IUI-51 |
| IUI-48 | **input into** | IUI-53 | at t57 | The misdiagnosis is input into the writing process |
| IUI-46 | **input into** | IUI-53 | at t58 | As is the cognitive representation |
| IUI-50 | **output of** | IUI-53 | at t59 | The sentence is output of the writing process |
