# Supplementary material for: Diagnosis, misdiagnosis, lucky guess, hearsay, and more: an ontological analysis
Source: J Biomed Semantics. 2016 Sep 15;7:54. doi: 10.1186/s13326-016-0098-5 (PMC5025551; doi:10.1186/s13326-016-0098-5)
Supplement: Additional file 4: Table S10. — Entities in Scenario 5: Layperson’s justified inference. Table S11. Additional temporal entities in Scenario 5: Layperson’s justified inference. Table S12. Relationships among particulars in Scenario 5: Layperson’s justified inference. (DOCX 92 kb) [file 13326_2016_98_MOESM4_ESM.docx]

**Table S10**. Entities in Scenario 5: *Layperson’s justified inference.*

| **IUI** | **Entity** | **Existence period** | **Type** | **Notes** |
| --- | --- | --- | --- | --- |
| IUI-83 | Mr. Jones’ daughter | t83 | Human being |  |
| IUI-84 | Cognitive system of IUI-83 | t84 |  |  |
| IUI-85 | An anatomical entity that is part of IUI-84 | t85 | Anatomical entity | Which anatomical entity and its lifetime cannot be easily specified given current state of neuroscience. |
| IUI-86 | Quality that inheres in IUI-85 and is about IUI-7 | t86 | Cognitive representation |  |
| IUI-7 | The POR that is truth-maker for IUI-28 | t7 | Configuration | Mr. Jones, his disease, their relationship, and disease’s instantiation |
| IUI-88 | The daughter’s justified conclusion | t88 | ICE | ICE concretized by IUI-86 & IUI-90 |
| IUI-89 | That which is written down on paper and forms the sentence | t89 | Material entity | *Dad has type 2 diabetes mellitus.* |
| IUI-90 | IQE that inheres in IUI-89 | t90 | Information quality entity | The sentence began to exist as soon as ink was laid down on paper, but the IQE did not begin to exist until the sentence was finished |
| IUI-91 | The daughter’s process of reasoning | occupies t91 | Process | The daughter’s process of reasoning that led to her conclusion that her father has type 2 diabetes mellitus |
| IUI-92 | The aggregate of Dr. Smith’s diagnosis and the daughter’s trust in Dr. Smith | t92 | Cognitive representation |  |
| IUI-93 | The daughter’s process of writing the sentence in her letter | occupies t93 | Process |  |

**Table S11.** Additional temporal entities in Scenario 5: *Layperson’s justified inference.*

| **Temporal identifier** | **Description** | **Notes** |
| --- | --- | --- |
| t94 | The interval during which the anatomical entity (IUI-85) is part of the cognitive system (IUI-84) | This interval is not easily specified given the current state of neuroscience. It could be different than t83 and t84. |
| t95 | The interval during which the clinical picture (IUI-92) is used in the interpretive process (IUI-91) | Could be shorter than t91 |
| t96 | The point in time at which the cognitive representation (IUI-86) and conclusion (IUI-88) begin to exist | t96 ends t91. Because the ICE does not exist until the cognitive representation—its first concretization—exists, this is also the point in time at which the conclusion begins to exist. |
| t97 | The interval during which the cognitive representation (IUI-86) participates in the writing process (IUI-93) |  |
| t98 | The interval during which the conclusion (IUI-88) participates in the writing process (IUI-93) | It is possible that the original cognitive representation (IUI-86) gets copied elsewhere in the brain for reasoning and thus that the ICE continues to participate after the initial cognitive representation. |
| t99 | The interval during which that which is written on paper (IUI-90) begins to exist until it exists in full | The writing process begins earlier than the time at which the sentence begins to exist: the author starts the process with getting a pen and paper, any preparation necessary (“clicking” the pen), etc. |

**Table S12.** Relationships among particulars in Scenario 5: *Layperson’s justified inference.*

| **IUI** | **Relation** | **IUI** | **When relation holds in reality** | **Notes** |
| --- | --- | --- | --- | --- |
| IUI-84 | **part of** | IUI-83 | at t84 |  |
| IUI-85 | **part of** | IUI-84 | at t85 | All anatomical components in which the cognitive representation inheres are part of the cognitive system. We do not assume the cognitive system is limited to the brain or even nervous system. |
| IUI-86 | **inheres in** | IUI-85 | at t86 |  |
| IUI-86 | **is about** | IUI-7 | at t86 | The cognitive representation stands in aboutness to IUI-7 as long as it exists |
| IUI-86 | **is about** | IUI-1 | at t86 | It is also about Mr. Jones |
| IUI-86 | **is about** | IUI-2 | at t86 | And about Mr. Jones’ disease |
| IUI-86 | **is about** | UUI-1 | at t86 | And about Type 2 diabetes mellitus |
| IUI-86 | **concretizes** | IUI-88 | at t86 | It also concretizes the conclusion |
| IUI-90 | **inheres in** | IUI-89 | at t89 | The IQE inheres in the sentence on paper |
| IUI-90 | **is about** | IUI-7 | at t90 | The IQE stands in aboutness to IUI-7 |
| IUI-90 | **is about** | IUI-1 | at t90 | It is also about Mr. Jones |
| IUI-90 | **is about** | IUI-2 | at t90 | And about Mr. Jones’ disease |
| IUI-90 | **is about** | UUI-1 | at t90 | And about Type 2 diabetes mellitus |
| IUI-90 | **concretizes** | IUI-88 | at t90 | The IQE concretizes the conclusion |
| IUI-90 | **is conformant to** | IUI-86 | at t90 | The IQE is conformant to the cognitive representation as long as it exists |
| IUI-83 | **agent in** | IUI-91 | at t91 |  |
| IUI-92 | **input into** | IUI-91 | at t95 | Cognitive representation input into IUI-91 |
| IUI-86 | **output of** | IUI-91 | at t96 | Cognitive representation output from IUI-91 |
| IUI-88 | **output of** | IUI-91 | at t96 | Both the conclusion and its concretization are outputs of IUI-91 |
| IUI-88 | **input into** | IUI-93 | at t97 | The conclusion is input into the writing process |
| IUI-86 | **input into** | IUI-93 | at t98 | As is the cognitive representation |
| IUI-90 | **output of** | IUI-93 | at t99 | The sentence is output of the writing process |
